# Metabolically Healthy or Metabolically Unhealthy Obese HIV-Infected Patients: Mostly a Matter of Age?

**DOI:** 10.3389/fendo.2018.00681

**Published:** 2018-11-16

**Authors:** João Sérgio Neves, Vanessa Guerreiro, Davide Carvalho, Rosário Serrão, António Sarmento, Paula Freitas

**Affiliations:** ^1^Department of Endocrinology, Diabetes and Metabolism, Centro Hospitalar Universitário de São João, EPE, Porto, Portugal; ^2^Departamento de Cirurgia e Fisiologia, Unidade de Investigação Cardiovascular, Faculdade de Medicina, Universidade do Porto, Porto, Portugal; ^3^Faculdade de Medicina, Instituto de Investigação e Inovação em Saúde, Universidade do Porto, Porto, Portugal; ^4^Infectious Diseases Department, Centro Hospitalar Universitário de São João, EPE, Porto, Portugal

**Keywords:** obesity, HIV, metabolic syndrome, metabolically healthy obesity, aging

## Abstract

**Background:** Life expectancy of HIV-infected patients has increased with antiretroviral treatment (ART). Chronic diseases associated with aging, including metabolic and cardiovascular diseases are becoming more prevalent in this population. We aimed to evaluate the association of obesity and aging with cardiometabolic comorbidities and metabolic health status among patients with HIV infection.

**Methods:** We evaluated 580 HIV-1 infected patients (71.7% male, mean age of 47.7 ± 11.5 years). We analyzed the association of age and obesity (defined by and by central obesity) with gender, duration of HIV infection, and ART, anthropometric parameters, cardiometabolic comorbidities, Framingham risk score (FRS), blood pressure, lipid profile, uric acid, liver biochemical tests, and glycemic profile. Furthermore, we analyzed the above-mentioned associations according to the category and central obesity into the metabolically healthy (MH) and unhealthy (MUH) categories. To evaluate the association of anthropometric parameters with cardiometabolic comorbidities, we performed unadjusted and adjusted logistic regression models.

**Results:** The prevalence of excessive weight and cardiometabolic comorbidities increased with age. Patients with normal weight were younger and there was a higher proportion of female patients in the obesity group. The prevalence of hypertension and metabolic syndrome were higher among patients who were overweight or with obesity. The FRS was higher among patients with obesity. The proportion of MUH patients was higher among patients with excessive weight and central obesity. MUH patients had more cardiometabolic comorbidities and a higher FRS. In the normal weight group, MUH patients were older, and in the obesity group they were more likely to be male. The anthropometric parameter most associated with metabolic syndrome was waist circumference and that most associated with hypertension was waist-to-height ratio. The anthropometric parameter most associated with diabetes and FRS was waist-to-hip ratio.

**Conclusion:** Patients with HIV present a high prevalence of obesity and related comorbidities. Ageing significantly contributes to metabolic dysfunction in this population. The proportion of MUH patients is higher among groups with excessive weight and central obesity, with those patients presenting a higher cardiovascular risk. Our results highlight the importance of evaluating and addressing obesity in patients with HIV, as well as metabolic comorbidities and cardiovascular risk.

## Introduction

With the introduction of combination antiretroviral therapy (cART), people living with human immunodeficiency virus (HIV) have experienced a dramatic improvement in life expectancy, which has changed the characteristics of HIV infection from a fatal disease to a chronic manageable infectious disease (1). Consequently, HIV-infected individuals are growing older and as a result, chronic diseases associated with aging are becoming more prevalent in this population, including metabolic diseases and cardiovascular diseases (2).

Contemporary ART is more effective and better-tolerated than older ART. Nonetheless, metabolic complications and downstream cardiovascular disease risk persist in people with HIV. Furthermore, ever since the introduction of cART, the prevalence of HIV–associated wasting has declined, while the proportion of overweight and obese HIV-infected individuals has increased (3–6).

It is to be expected that, similar to the general population, obesity rates also increase with growing older in HIV-infected patients (7). Currently, we are in the midst of several demographic shifts among HIV-infected patients, with aging and obesity becoming equally more prevalent (8). HIV-infected patients who are overweight and with obesity have a higher prevalence of multi-morbidity than normal or underweight patients (8). In the past studies, the methodology of definition of obesity has proved to be another aspect of primordial relevance. Although the majority of studies have used BMI as the general obesity index, central obesity may have higher predictive value with regard to health problems than BMI, especially cardiometabolic ones (9, 10). Waist circumference predicts obesity-related health risk, as waist circumference measurement is a substitute marker of visceral adiposity, and accordingly, waist circumference has been established as being an easy tool for assessing abdominal obesity (11).

Obesity is generally associated with the development of metabolic disorders, however, the manifestation of obesity without overt cardiometabolic disease has been described as “metabolically healthy obesity.” The prevalence of metabolically healthy obesity ranges from 6 to 40%, depending on the different populations studied (12–15). The existence of metabolically healthy obesity is controversial, both in uninfected and infected HIV-patients, but is supported by the fact that metabolic dysregulation is a heterogeneous process that also occurs in the absence of obesity. People with metabolically healthy obesity may have less visceral adipose tissue and systemic inflammation, together with more favorable immune profiles than metabolically unhealthy obese people (16–18), which further supports a spectrum of metabolic health within obesity. In contrast, some studies have suggested the presence of increased mortality and increased CVD risk according to differences in body composition and fat distribution in HIV-uninfected people who meet the criteria for metabolically healthy obesity, suggesting that this state is not entirely benign, or may not even exist (15, 19–23). This doubt also exists for the HIV-infected population. It is not known whether metabolically healthy obesity exists in HIV-infected patients. Given the prevalence of metabolic syndrome in HIV (representing up to 45 vs. 25% for the general population) (24), as well as the metabolic effects of HIV and cART and the persistent immune activation and inflammation associated with even virologically-suppressed HIV (25), it is possible that metabolically healthy obesity may not exist in HIV-infected patients at all, and/or that metabolic health in this population must be defined differently (26).

From a clinical point of view, the body mass index (BMI) is an easy and non-invasive anthropometric parameter which is more frequently used in clinical settings, due to its comparability across different populations and areas (27). BMI permits the classification of patients as being overweight or obese and also the prediction of their cardio-metabolic risk (28, 27). However, the use of BMI in ascertaining cardio-metabolic risk is not without drawbacks, as several other factors are not accounted for, such as important muscle mass, age, and ethnicity (11, 27). For example, a patient with a high BMI resulting from significant muscle mass may not necessarily have a high body fat mass, which is a better predictor of cardio-metabolic risk. On the contrary, patients with abdominal obesity resulting from intra-abdominal visceral adiposity do not always have elevated BMI (29), although their cardiometabolic risk is high. Waist circumference, waist-to-hip ratio, and waist-to-height ratio are all independent predictors of cardio-metabolic risk and therefore could be important tools for assessing increased visceral adiposity in HIV-infected patients on antiretroviral therapy (30–32). Waist circumference is a key parameter which is often used to define central obesity, as it is a parameter of the definition of metabolic syndrome (33) and is significantly associated with the other parameters of the metabolic syndrome (34). Waist-to-hip ratio, which is an even more accurate measure of central adiposity and a predictor of cardio-metabolic risk (35) has been recommended by the World Health Organization (WHO) for defining metabolic syndrome (36). Consequently, the waist-to-height ratio was reported to be a better predictor of cardiometabolic risk than BMI in a review by Savva et al. (37). However it is defined, obesity contributes to cardiometabolic disease risk (dyslipidemia, hypertension, diabetes, cardiovascular risk) among people who are not infected with HIV (38), and it can significantly impact the disease burden among those who are affected with HIV (8, 39, 40). HIV and obesity are pro-inflammatory conditions which, when occurring together, can pose a synergistic risk for metabolic and cardiovascular disease. However, we need to know more about this association of obesity with age and cardiometabolic abnormalities in HIV-infected patients undergoing cART.

The aims of this study were the following: (1) to evaluate how obesity and associated metabolic comorbidities progress according to age; (2) to evaluate the prevalence of obesity as defined by BMI and by central obesity, and also the prevalence of metabolic alterations and CV risk (evaluated by the 10 year Framingham risk score according to these two definitions of obesity); (3) to evaluate whether there are metabolically healthy people among the subpopulations of normal weight, overweight, and obese individuals, as defined by BMI; (4) to evaluate whether there are metabolically healthy individuals according to the presence, or absence of central obesity, and; (5) to evaluate how the parameters that evaluate obesity (BMI, waist circumference, waist-to-hip ratio, waist-to-weight ratio) correlate with metabolic changes and CV risk in an HIV-infected population under combined antiretroviral therapy.

## Methods

### Subjects

As part of a cross-sectional study, 610 Caucasian, non-institutionalized, HIV-1 infected and cART-treated adult outpatients who were being followed at Hospital São João, Porto, were consecutively evaluated. The study agreement was approved by the Hospital Ethics Committee, and all the patients provided a signed informed consent form. Patients with underweight (BMI < 18.5 kg/m^2^) were excluded from our analysis.

### Clinical assessment

For each patient the following data were collected, using a standardized protocol: demographic data (age, gender), duration of HIV infection, and; duration of cART. Weight, height, and circumferences of waist and hip were also measured. Body weight was measured using the TANITA (Tanita®, model TBF 300) scale, with patients being obliged to wear light clothes without shoes, and height was measured to the nearest centimeter in the standing position, using a wall stadiometer (Holtain Limited Crymych, Dyfed®).

Hip circumference was measured, with the patient standing erect with arms by their side, with their feet placed together and the gluteal muscles relaxed. An inelastic tape was placed around the buttocks in a horizontal plane. To ensure continuity of the two parts of the tape used to measure the circumference, the cross-handed technique was used, whereby an assistant checked the position of the tape on the opposite side of the patient's body. The tape was in contact with the skin, but it did not compress the soft tissues. Waist circumference was measured at the end of a gentle expiration of a breath, midway between the lowest rib and iliac crest, with the patient standing up. Hip circumference were measured at the greater trochanter, with the patient standing upright, facing forward, and their shoulders relaxed. All measurers were performed by the same observer using standard techniques (41). Resting blood pressure (BP) was taken whilst in a supine position, and was measured in a standard fashion, as previously described (24).

### Laboratory analysis

A venous blood sample was taken after a 12-h overnight fast, and all the samples were analyzed at the central laboratory of our hospital. Serum levels were determined for the following, using commercial kits: total cholesterol (TC); low-density lipoprotein (LDL) cholesterol; high-density lipoprotein (HDL) cholesterol; triglycerides; uric acid; creatinine; alanine aminotransferase (AST); alanine aminotransferase (ALT); γ-glutamyl transferase (GGT); alkaline phosphatase; glucose; insulin, and; glycated hemoglobin (HbA1c). Estimated glomerular filtration rate (eGFR) was calculated using the Chronic Kidney Disease Epidemiology Collaboration (CKD-EPI) equation (42). Insulin resistance was defined by the homeostasis model assessment of insulin resistance (HOMA-IR), using the following formula: (fasting insulin x fasting glucose)/22.5 (43). The CD4+ cell count was determined by flow cytometry, and plasma HIV-1 RNA loads were measured by a quantitative reverse transcriptase polymerase chain reaction, which has a lower limit of detection of 50 copies/mL, as performed in routine clinical care.

### Definition of metabolic status, cardiometabolic comorbidities, and cardiovascular risk score

The metabolic status was defined by the presence, or absence of metabolic syndrome, according to the harmonized International Diabetes Federation criteria (44), which required the presence of three of the following risk factors: (1) elevated waist circumference (≥80 cm in women and ≥94 cm in men); (2) elevated triglycerides (≥150 ml/dL or specific treatment); (3) reduced HDL-C levels (< 50 mg/dL in women and < 40 mg/dL in men), (4) elevated blood pressure (>130/ 85 mmHg or treatment with anti-hypertensive drugs), and; (5) elevated fasting glucose (≥100 mg/dL or use of glucose-lowering drugs). Patients that did not manifest criteria for metabolic syndrome were classified as being metabolically healthy, whereas those patients with metabolic syndrome were classified as being metabolically unhealthy (45).

Diabetes was defined by a previous diagnosis, use of antihyperglycemic drugs, fasting plasma glucose ≥126 mg/dL or HbA1c ≥6.5%. Prediabetes was defined by fasting plasma glucose ≥100 and < 126 mg/dL or HbA1c ≥5.7 and < 6.5% and hypertension was defined as systolic BP ≥140 mmHg, diastolic BP ≥90 mmHg, or the use of antihypertensive drugs. Dyslipidemia was defined by the use of lipid-lowering agents, serum LDL ≥100 mg/dL, serum HDL < 50 mg/dL in women or < 40 mg/dL in men, or serum triglycerides ≥150 mg/dL (46). Cardiovascular disease (CVD) risk at 10 years was measured by the Framingham Risk Score, using a sex-specific algorithm that incorporates age, treated and untreated systolic blood pressure, HDL-C, total cholesterol, glucose, and smoking status (47).

A high waist circumference was defined as ≥80 cm in women, and ≥94 cm in men (44), with a high waist-to-hip ratio being defined as a ratio ≥0.85 in women and ≥0.90 in men (48). A high waist-to-height ratio was defined as a ratio ≥0.50 in women and men (49).

### Statistical analysis

We performed the following analyses: (1) comparison of the prevalence of comorbidities between age groups (< 40 years, 40–64 years, and ≥65 years); (2) comparison of clinical and laboratory characteristics of patients, according to BMI category (normal weight, overweight, and obesity); (3) comparison of clinical and laboratory characteristics of patients according to waist circumference (normal waist circumference and central obesity); (4) comparison of clinical and laboratory characteristics of patients according to metabolic status (metabolically healthy or metabolically unhealthy) among groups of BMI category and waist circumference. Finally, (5) we evaluated the association of anthropometric parameters with cardiometabolic comorbidities, using unadjusted and adjusted logistic regression models. Model 1 includes gender and age, Model 2 includes gender, age, and ART duration, and Model 3 includes gender, age, ART duration, and BMI. Our sample size calculation was based on comparison of the prevalence of comorbidities between two subgroups. We calculated that to detect a difference of 20% in the prevalence of a comorbidity between two subgroups (with a prevalence of 10% in the control group), with a power of 80% and a significance level of 5%, a sample size of 62 patients in each subgroup would be needed. Results are presented as mean ± standard deviation for normally distributed continuous variables, a median (25th percentile – 75th percentile) for non-normally distributed continuous variables, and as percentages for categorical variables. For normally-distributed continuous variables, we used independent *t*-tests for comparison between two groups and analysis of variance (ANOVA) for comparison between multiple groups. For non-normally distributed continuous variables, we used Mann–Whitney *U*-tests for comparison between two groups, and Kruskal-Wallis tests for comparison between multiple groups. Differences between groups regarding categorical variables were evaluated with a chi-squared test. Statistical analyses were performed with Stata software, version 14.1 (StataCorp). We considered a two-sided P value less than 0.05 to be statistically significant.

## Results

### Characteristics of the study population

580 patients were included in our analysis. 30 underweight patients were excluded. 71.7% of the patients were male, the mean age was 47.7 ± 11.5 years old, and the median duration of HIV infection was 7 years (Table 1). 91.6% of the patients were treated with nucleoside reverse transcriptase inhibitors (NRTI), 45.2% with non-nucleoside reverse transcriptase inhibitors (NNRTI), and 48.6% with protease inhibitors (PI). 87% had HIV-1 RNA loads < 50 copies/ml. The mean BMI was 26.5 ± 4.8 kg/m^2^, and the mean waist circumference was 94.9 ± 11.8 cm. 24.4% had diabetes, 64.5% had hypertension, and 95.7% had dyslipidemia. 58.8% satisfied the criteria for metabolic syndrome, being classified as metabolically unhealthy.

**Table 1:** Clinical characteristics of the population according to BMI category.

	**Total (*n* = 580)**	**Normal weight (*n* = 241)**	**Overweight (*n* = 216)**	**Obesity (*n* = 123)**	***P*-value**
Age, years	47.7 ± 11.5	45.5 ± 11.1	49.8 ± 11.5	48.4 ± 11.2	**<0.001**
Male sex, %	71.7%	75.1%	73.1%	62.6%	**0.037**
Years of HIV infection	7 (3–10)	8 (4–11)	6 (3–10)	5.5 (2–9)	0.089
Years of ART	8 (5–12)	9 (6–12)	7 (4–11)	8 (5–12)	**0.007**
ART, %				
PI	48.6%	52.9%	49.0%	39.3%	0.053
NNRTI	45.2%	46.1%	43.2%	47.0%	0.752
NRTI	91.6%	95.3%	90.8%	85.5%	**0.007**
CD4 cell count, cells/mm3	485 (338–691)	468 (334–681)	484 (327–719)	529 (366–715)	0.374
HIV RNA (< 50), %	87.0%	88.4%	86.3%	85.1%	0.642
Body mass index, kg/m^2^	26.5 ± 4.8	22.3 ± 1.7	27.1 ± 1.4	33.8 ± 3.5	**<0.001**
Waist circumference, cm	94.9 ± 11.8	85.5 ± 6.7	97.3 ± 7.0	110.2 ± 9.0	**<0.001**
Hip circumference, cm	96.7 ± 9.0	90.7 ± 4.9	97.7 ± 6.0	108.2 ± 9.1	**<0.001**
Waist-to-hip ratio	0.98 ± 0.08	0.94 ± 0.07	1.00 ± 0.08	1.02 ± 0.09	**<0.001**
Waist-to-height ratio	0.57 ± 0.07	0.51 ± 0.04	0.59 ± 0.04	0.67 ± 0.06	**<0.001**
Diabetes mellitus, %	24.4%	21.2%	25.0%	29.5%	0.208
Prediabetes, %	20.3%	16.1%	21.2%	26.8%	0.053
Hypertension, %	64.5%	54.8%	73.1%	68.6%	**<0.001**
Dyslipidemia, %	95.7%	95.4%	95.8%	95.9%	0.970
Metabolic syndrome, %	58.8%	39.8%	71.0%	75.2%	**<0.001**
Framingham 10-y risk, %	9.9 (4.6–21.0)	8.1 (3.8–17.5)	12.1 (5.6–22.7)	10.3 (4.8–21.9)	**0.004**
Systolic BP, mmHg	125.1 ± 18.6	119.7 ± 18.4	127.6 ± 16.0	131.4 ± 20.6	**<0.001**
Diastolic BP, mmHg	76.4 ± 11.4	74.2 ± 11.6	76.9 ± 10.1	80.1 ± 12.1	**<0.001**
Total cholesterol, mg/dl	216.6 ± 56.3	212.3 ± 56.7	221.7 ± 60.1	216.3 ± 47.1	0.203
LDL cholesterol, mg/dl	130.6 ± 46.0	126.7 ± 45.4	135.4 ± 47.7	130.1 ± 43.9	0.132
HDL cholesterol, mg/dl	46.5 ± 13.5	46.5 ± 13.9	46.3 ± 13.2	46.9 ± 13.5	0.926
Triglycerides, mg/dl	182 (123–282)	184 (116–300)	187 (130–271)	178 (110–263)	0.797
eGFR, ml/min/1.73m^2^	93.4 ± 17.2	95.2 ± 18.1	91.3 ± 17.0	93.2 ± 14.8	0.091
Uric acid, mg/dl	5.0 ± 1.6	4.7 ± 1.4	5.2 ± 1.8	5.5 ± 1.6	**<0.001**
AST, U/L	25 (20–36)	25 (20–39)	25 (20–35)	23 (20–34)	0.441
ALT, U/L	27 (19–45)	26 (19–44)	28 (19–46)	27 (21–43)	0.937
GGT, U/L	44 (28–85)	47 (28–96)	44 (26–84)	41 (27–74)	0.432
Alkaline phosphatase, U/L	85 (65–105)	87 (71–107)	80 (62–101)	85 (67–105)	**0.015**
Glucose, mg/dl	95 (86–116)	92 (84–107)	80 (62–101)	101 (90–118)	**0.001**
HbA1c, mg/dl	5.7 ± 1.1	5.6 ± 1.4	5.7 ± 1.0	5.7 ± 0.8	0.952
HOMA-IR, mg/dl	2.1 (1.2–4.0)	1.8 (1.1–3.5)	2.5 (1.4–4.0)	2.5 (1.3–5.7)	0.952

ART, antiretroviral therapy; PI, protease inhibitors; NNRTI, non-nucleoside reverse transcriptase inhibitors; NRTI, nucleoside reverse transcriptase inhibitors; BP, blood pressure; LDL, low density lipoproteins; HDL, high density lipoproteins; eGFR, estimated glomerular filtration rate; AST, aspartate transaminase; ALT, alanine transaminase; GGT, gamma-glutamyl transferase; HOMA-IR, homeostatic model assessment of insulin resistance.

*The values highlighted in bold are statistically significant (P < 0.05)*.

### Obesity and associated metabolic comorbidities according to age

**Figure 1:**
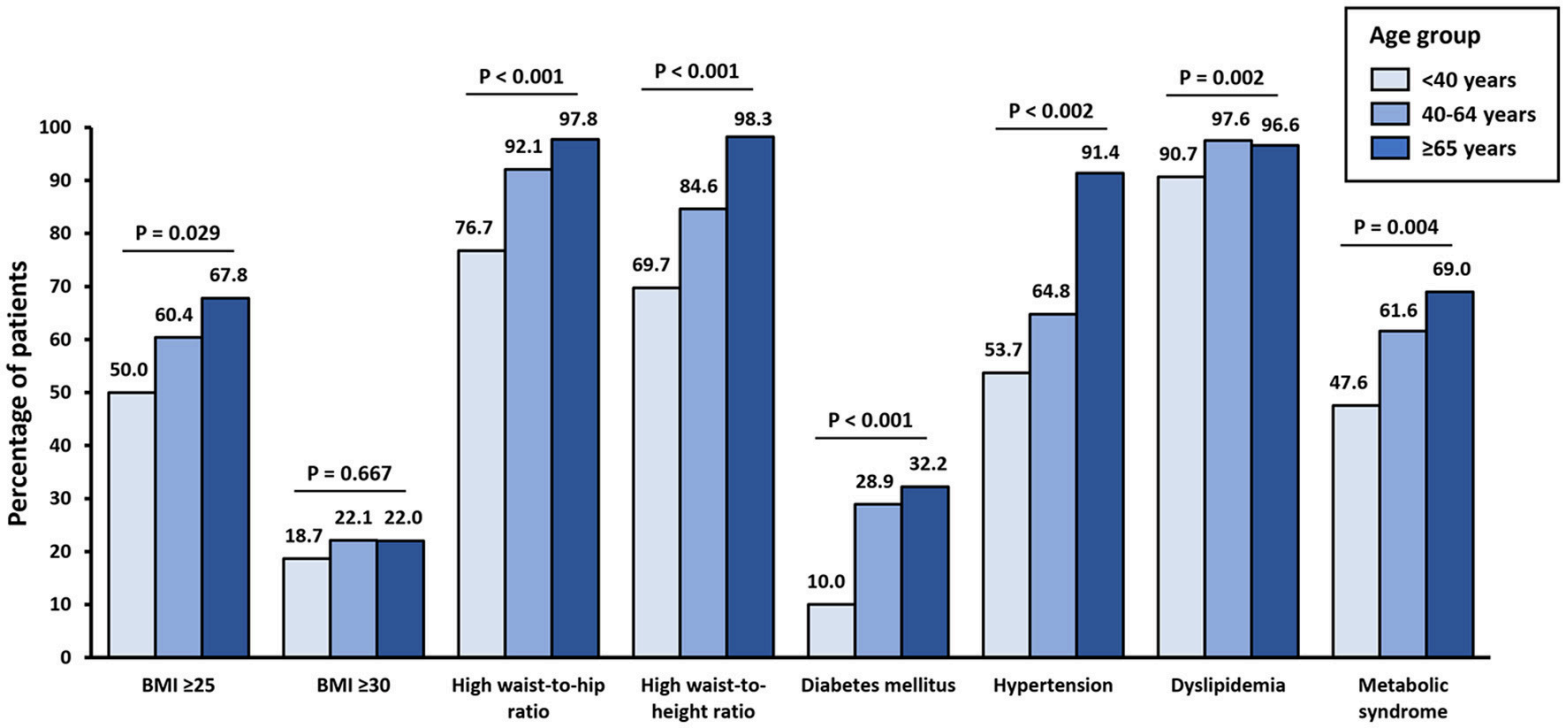
Prevalence of being overweight, obesity, high waist-to-hip ratio, high waist-to-height ratio, diabetes mellitus, hypertension, and metabolic syndrome according to age group. BMI, body mass index (kg/m^2^).

The prevalence of overweight and obesity (BMI ≥ 25 kg/m2) increased significantly with age category (< 40 years: 50.0%, 40–64 years: 60.4% and ≥65 years: 67.8%; *p* = 0.029). The prevalence of high waist-to-hip ratio (≥0.85 in women and ≥0.90 in men) and high waist-to-height ratio (≥0.50 in women and men) also increased significantly with age. Regarding the metabolic comorbidities associated with obesity, there was a significant and progressive increase in the prevalence of diabetes, hypertension, and metabolic syndrome. Dyslipidemia was also significantly more prevalent among patients who were 40–64 years old, or ≥65 years old, when compared with patients < 40 years (Figure 1).

### Clinical characteristics of the population according to BMI category and central obesity

BMI category was significantly associated with several clinical characteristics (Table 1). For example, patients in the normal weight group were younger and there was a lower proportion of male patients in the obesity group. While the duration of HIV infection was similar between groups, patients in the normal weight group had a longer median period of ART. The CD4 cell count and the proportion of patients with HIV-1 RNA loads < 50 copies/mL was similar between groups. The prevalence of hypertension and metabolic syndrome was higher among patients who were overweight or with obesity, when compared with patients with normal weight. The mean systolic and diastolic BP were also higher among patients who were overweight or with obesity, when compared with patients with normal weight. Alkaline phosphatase was lower in patients who were overweight, when compared with patients with normal weight or with obesity. Both uric acid levels and fasting plasma glucose levels, presented a significant progressive increase from the normal weight category to the obesity category. The Framingham 10 years risk score was significantly higher among patients who were overweight or with obesity, when compared with patients with normal weight.

Patients with central obesity were older than patients with normal waist circumference, and there was a higher proportion of female patients in the central obesity group. The median duration of ART treatment was lower in the central obesity group, and there was a lower proportion of participants treated with NRTI. Participants with central obesity had a significantly higher prevalence of prediabetes, hypertension, and metabolic syndrome. The Framingham 10 years risk score was significantly higher among patients with central obesity. Patients in the central obesity group also presented higher systolic and diastolic BP, and higher total cholesterol, LDL cholesterol and HDL cholesterol. eGFR was significantly lower in the central obesity group, while AST and ALT were higher in the normal waist group. Both fasting plasma glucose and HOMA-IR were significantly higher in patients with central obesity (Table 2).

**Table 2:** Clinical characteristics of the population according to central obesity.

	**Normal waist circumference (*n* = 215)**	**Central obesity (*n* = 335)**	***P*-value**
Age, years	44.5 ± 10.5	49.8 ± 11.8	**<0.001**
Male sex, %	90.7%	61.2%	**<0.001**
Years of HIV infection	7 (4–11)	6 (3–10)	0.067
Years of ART	9 (6–12)	7 (4–11)	**0.047**
ART, %		
PI	52.4%	46.6%	0.191
NNRTI	47.6%	43.8%	0.390
NRTI	95.1%	90.4%	**0.043**
CD4 cell count, cells/mm3	465 (332–672)	487 (338–719)	0.363
HIV RNA (< 50), %	88.9%	85.5%	0.258
Body mass index, kg/m^2^	22.9 ± 2.4	28.6 ± 4.4	**<0.001**
Waist circumference, cm	84.9 ± 5.9	101.3 ± 10.1	**<0.001**
Hip circumference, cm	91.0 ± 5.0	100.6 ± 9.0	**<0.001**
Waist-to-hip ratio	0.94 ± 0.07	1.01 ± 0.08	**<0.001**
Waist-to-height ratio	0.51 ± 0.04	0.61 ± 0.06	**<0.001**
Diabetes mellitus, %	21.4%	27.5%	0.109
Prediabetes, %	15.8%	23.0%	**0.041**
Hypertension, %	56.1%	69.8%	**0.001**
Dyslipidemia, %	94.9%	95.8%	0.607
Metabolic syndrome, %	31.6%	77.2%	**<0.001**
Framingham 10-y risk, %	8.4 (4.0–17.5)	11.2 (4.9–23.0)	**0.009**
Systolic BP, mmHg	120.0 ± 17.2	128.2 ± 18.8	**<0.001**
Diastolic BP, mmHg	74.2 ± 11.2	77.8 ± 11.2	**<0.001**
Total cholesterol, mg/dl	210.4 ± 56.6	222.1 ± 56.2	**0.018**
LDL cholesterol, mg/dl	125.1 ± 45.7	134.8 ± 46.5	**0.017**
HDL cholesterol, mg/dl	44.9 ± 13.2	47.4 ± 13.2	**0.029**
Triglycerides, mg/dl	196 (124–301)	182 (128–280)	0.725
eGFR, ml/min/1.73m^2^	96.7 ± 16.5	90.9 ± 17.3	**<0.001**
Uric acid, mg/dl	4.9 ± 1.4	5.1 ± 1.7	0.316
AST, U/L	28 (21–43)	23 (19–33)	**<0.001**
ALT, U/L	31 (19–55)	25 (19–40)	**0.025**
GGT, U/L	48 (29–105)	43 (26–77)	0.068
Alkaline phosphatase, U/L	85 (66–105)	84 (64–105)	0.572
Glucose, mg/dl	92 (84–112)	99 (88–118)	**0.002**
HbA1c, mg/dl	5.6 ± 1.4	5.6 ± 0.9	0.5732
HOMA-IR, mg/dl	1.8 (1.1–3.2)	2.5 (1.3–4.3)	**0.013**

Patients were classified as having central obesity if waist circumference was ≥80 cm in women, or ≥94 cm in men. ART, antiretroviral therapy; PI, protease inhibitors; NNRTI, non-nucleoside reverse transcriptase inhibitors; NRTI, nucleoside reverse transcriptase inhibitors; BP, blood pressure; LDL, low density lipoproteins; HDL, high density lipoproteins; eGFR, estimated glomerular filtration rate; AST, aspartate transaminase; ALT, alanine transaminase; GGT, gamma-glutamyl transferase; HOMA-IR, homeostatic model assessment of insulin resistance.

*The values highlighted in bold are statistically significant (P < 0.05)*.

### Metabolic healthy and unhealthy patients according to BMI category

While most patients in the normal BMI group were metabolically healthy (39.8% metabolically unhealthy participants in the normal BMI group), most of the patients in the overweight and obesity groups were classified as being metabolically unhealthy (71.0 and 75.2% respectively). The differences between metabolic healthy and metabolic unhealthy patients within each category of BMI are presented in Table 3. Among patients with normal BMI, metabolically unhealthy patients were older and had a longer duration of HIV infection. The prescription of NNRTI was lower for metabolically unhealthy patients with normal BMI. On the other hand, there were no differences in the age, sex, and HIV duration or treatment between metabolically healthy and metabolically unhealthy patients in the overweight group. In the group with obesity, the proportion of male patients was significantly higher among the metabolically unhealthy subgroup. For both the normal weight and overweight groups, metabolically unhealthy patients presented higher waist circumference, and higher waist-to-hip ratio and waist-to-height ratio. In the obesity group, metabolically unhealthy participants had a lower hip circumference and a higher waist-to-hip ratio. In all BMI categories, metabolically unhealthy patients had a higher prevalence of diabetes, hypertension, and dyslipidemia, with a significantly increased Framingham 10 years risk score. Systolic and diastolic BP were also higher in metabolically unhealthy patients in all categories of BMI. HDL cholesterol and triglycerides were higher among metabolically unhealthy patients in all categories of BMI, while LDL was lower in the metabolically unhealthy participants in the obesity category. eGFR was lower in metabolically unhealthy patients in the normal weight group, while uric acid was higher in metabolically unhealthy patients in the overweight group. GGT was higher in the metabolically unhealthy patients in the overweight and obesity category, while alkaline phosphatase was lower in metabolically unhealthy patients in the obesity category. HOMA-IR was higher in metabolically unhealthy participants of the overweight and obesity categories, and fasting plasma glucose and HbA1c were higher in metabolically unhealthy participants in all categories of BMI.

**Table 3:** Comparison of metabolic healthy and unhealthy patients within each category of BMI.

	**Normal weight**		**Overweight**		**Obesity**	
	**MH (*n* = 142)**	**MUH (*n* = 94)**	**MH (= 61)**	**MUH (*n* = 149)**	**MH (*n* = 29)**	**MUH (*n* = 88)**
Age, years	43.0 ± 10.4	48.8 ± 11.3^*^	48.8 ± 11.6	50.4 ± 11.4	50.3 ± 12.7	48.6 ± 10.5
Male sex, %	76.1%	74.4%	68.9%	75.1%	41.4%	70.5%^*^
Years of HIV infection	9 (5–11)	10 (7–13)^*^	6 (4–11)	7 (4–11)	9 (7–12)	7 (4–11)
Years of ART	7 (3–10)	8 (4–12)	6 (2–10)	7 (3–10)	7 (5–10)	4 (2–9)
ART, %					
PI	48.2%	61.3%	45.5%	50.3%	44.4%	38.1%
NNRTI	52.6%	36.6%^*^	47.3%	42.8%	55.5%	47.6%
NRTI	97.1%	92.5%	92.7%	91.0%	88.8%	86.9%
CD4 cell count, cells/mm3	485 (359–689)	442 (289–641)	457 (314–682)	486 (329–749)	530 (372–662)	523 (366–716)
HIV RNA (< 50), %	90.5%	84.9%	92.7%	84.7%	96.2%	84.1%
Body mass index, kg/m^2^	22.2 ± 1.7	22.5 ± 1.8	27.2 ± 1.5	27.1 ± 1.4	33.7 ± 3.3	33.5 ± 3.3
Waist circumference, cm	83.3 ± 5.9	88.6 ± 6.6^*^	94.7 ± 7.9	98.4 ± 6.3^*^	109.0 ± 7.0	110.5 ± 9.6
Hip circumference, cm	90.6 ± 5.0	90.9 ± 4.8	98.9 ± 6.7	97.2 ± 5.7	113.0 ± 11.8	106.7 ± 7.9^*^
Waist-to-hip ratio	0.92 ± 0.07	0.97 ± 0.07^*^	0.96 ± 0.08	1.01 ± 0.07^*^	0.97 ± 0.11	1.03 ± 0.08^*^
Waist-to-height ratio	0.50 ± 0.04	0.53 ± 0.04^*^	0.58 ± 0.04	0.59 ± 0.04^*^	0.67 ± 0.04	0.67 ± 0.06
Diabetes mellitus, %	13.4%	33.0%^*^	1.6%	35.6%^*^	0%	40.9%^*^
Prediabetes, %	8.5%	26.7%^*^	8.2%	27.5%^*^	20.7%	29.5%
Hypertension, %	36.6%	82.6%^*^	47.5%	82.6%^*^	24.1%	83.9%^*^
Dyslipidemia, %	93.0%	98.9%^*^	90.2%	99.3%^*^	93.1%	100%^*^
Framingham 10-y risk, %	6.3 (2.9–12.7)	12.0 (6.2–23.4)^*^	8.0 (4.2–14.5)	15.9 (7.1–25.9)^*^	5.3 (2.4–8.4)	13.8 (6.2–26.9)^*^
Systolic BP, mmHg	115.1 ± 17.3	127.1 ± 17.2^*^	123.8 ± 16.8	128.7 ± 15.7^*^	120.3 ± 18.4	135.2 ± 20.0^*^
Diastolic BP, mmHg	71.4 ± 11.4	78.8 ± 10.0^*^	73.4 ± 9.3	78.2 ± 10.1^*^	73.7 ± 12.1	82.9 ± 11.1^*^
Total cholesterol, mg/dl	213.0 ± 54.9	213.4 ± 59.6	233.4 ± 65.5	217.4 ± 57.5	224.3 ± 34.5	214.7 ± 50.4
LDL cholesterol, mg/dl	129.8 ± 44.3	122.9 ± 47.5	145.6 ± 41.9	131.8 ± 49.5	147.5 ± 45.8	125.1 ± 42.0^*^
HDL cholesterol, mg/dl	49.4 ± 12.9	42.6 ± 14.5^*^	54.0 ± 14.4	43.1 ± 11.4^*^	56.0 ± 9.3	43.9 ± 13.5^*^
Triglycerides, mg/dl	135 (103–223)	252 (184–359)^*^	130 (87–194)	198 (152–312)^*^	120 (92–167)	205 (141–321)^*^
eGFR, ml/min/1.73m^2^	97.7 ± 16.7	91.4 ± 19.8^*^	90.8 ± 15.8	91.5 ± 17.4	94.5 ± 9.7	92.5 ± 16.1
Uric acid, mg/dl	4.6 ± 1.3	4.8 ± 1.5	4.4 ± 1.4	5.5 ± 1.8^*^	4.9 ± 0.7	5.6 ± 1.7
AST, U/L	26 (20–39)	25 (21–39)	25 (18–33)	25 (20–36)	21 (20–24)	25 (20–36)
ALT, U/L	24 (17–48)	28 (21–43)	23 (16–43)	29 (21–46)	22 (19–29)	28 (22–47)
GGT, U/L	41 (27–107)	55 (32–96)	33 (24–62)	50 (28–85)^*^	30 (23–38)	47 (30–91)^*^
Alkaline phosphatase, U/L	86 (66–105)	87 (72–120)	88 (63–110)	77 (59–99)	101 (80–116)	82 (63–100)^*^
Glucose, mg/dl	90 (83–96)	102 (91–130)^*^	90 (83–94)	108 (93–131)^*^	89 (82–99)	108 (95–126)^*^
HbA1c, mg/dl	5.4 ± 1.3	5.9 ± 1.5^*^	5.3 ± 0.8	5.8 ± 1.0^*^	5.3 ± 0.5	5.8 ± 0.8^*^
HOMA-IR, mg/dl	1.5 (1.1–2.7)	2.5 (1.4–4.3)^*^	1.6 (1.2–2.2)	3.1 (1.6–5.1)^*^	2.0 (1.0–2.4)	3.1 (1.3–5.8)

*MH, metabolically healthy (absence of metabolic syndrome); MH, metabolically health (absence of metabolic syndrome); MUH, metabolically unhealthy (presence of metabolic syndrome); ART, antiretroviral therapy; PI, protease inhibitors; NNRTI, non-nucleoside reverse transcriptase inhibitors; NRTI, nucleoside reverse transcriptase inhibitors; BP, blood pressure; LDL, low density lipoproteins; HDL, high density lipoproteins; eGFR, estimated glomerular filtration rate; AST, aspartate transaminase; ALT, alanine transaminase; GGT, gamma-glutamyl transferase; HOMA-IR, homeostatic model assessment of insulin resistance*.

* *p < 0.05 vs. MH*.

### Metabolically healthy and metabolically unhealthy patients with, and without central obesity

The proportion of metabolically unhealthy patients was significantly higher in the group with central obesity, when compared with participants with normal waist circumference (77.2 vs. 31.6%, *p* < 0.001). The differences between metabolically healthy and metabolically unhealthy patients according to the presence, or absence of central obesity are presented in Table 4. Both in the normal waist circumference group and in the central obesity group, metabolically unhealthy participants were more likely to be male and to have a higher waist circumference and waist-to-hip circumference. Furthermore, duration of HIV infection and ART were similar between groups, although there was a lower proportion of patients with HIV-1 RNA loads < 50 copies/mL among metabolically unhealthy participants in the central obesity category. Both in patients with, and without central obesity, metabolically unhealthy patients had a higher prevalence of diabetes, prediabetes, hypertension, and dyslipidemia. Metabolically unhealthy participants had higher systolic and diastolic BP, higher uric acid, higher LDL cholesterol, and triglycerides levels with lower HDL cholesterol in both groups. Glucose and HbA1c were higher in metabolically unhealthy patients of both groups, and HOMA-IR was higher in metabolically unhealthy patients of the central obesity group. The Framingham 10 years risk score was significantly increased in metabolically unhealthy patients with, and without central obesity, whereas in the normal waist circumference group, metabolically unhealthy patients had higher ALT levels, in the central obesity group metabolically unhealthy patients had increased levels of AST, ALT, and GGT.

**Table 4:** Comparison of metabolic healthy and unhealthy patients with and without central obesity.

	**Normal waist circumference**		**Central obesity**	
	**MH (*n* = 145)**	**MUH (*n* = 67)**	**MH (= 75)**	**MUH (*n* = 254)**
Age, years	43.6 ± 10.7	46.0 ± 9.3	49.1 ± 12.5	50.3 ± 11.4
Male sex, %	87.5%	97.0%^*^	40.0%	67.7%^*^
Years of HIV infection	8 (5–11)	10 (7–13)	7 (5–11)	8 (4–12)
Years of ART	6 (3–10)	8 (5–11)	6 (3–9)	6 (3–10)
ART, %			
PI	50.4%	55.2%	37.7%	49.8%
NNRTI	51.1%	41.8%	53.6%	41.7%
NRTI	96.4%	92.5%	95.7%	89.9%
CD4 cell count, cells/mm3	479 (351–682)	435 (289-632)	473 (309–676)	487 (343–724)
HIV RNA (< 50), %	90.6%	85.1%	94.1%	84.0%^*^
Body mass index, kg/m^2^	22.7 ± 2.3	23.3 ± 2.7	28.8 ± 4.6	28.5 ± 4.3
Waist circumference, cm	83.9 ± 6.1	87.0 ± 4.9^*^	99.2 ± 10.6	101.8 ± 9.9^*^
Hip circumference, cm	90.8 ± 5.1	91.2 ± 4.9^*^	103.4 ± 10.9	99.6 ± 8.2^*^
Waist-to-hip ratio	0.93 ± 0.07	0.96 ± 0.05^*^	0.96 ± 0.09	1.02 ± 0.08^*^
Waist-to-height ratio	0.50 ± 0.04	0.52 ± 0.03^*^	0.61 ± 0.06	0.62 ± 0.06
Diabetes mellitus, %	13.1%	38.8%^*^	1.3%	35.8%^*^
Prediabetes, %	8.3%	32.8%^*^	14.7%	26.0%^*^
Hypertension, %	40.7%	87.9%^*^	32.0%	81.0%^*^
Dyslipidemia, %	92.4%	100%^*^	90.6%	99.2%^*^
Framingham 10-y risk, %	6.5 (3.6–13.9)	13.3 (7.7–23.3)^*^	5.7 (2.3–13.6)	13.5 (6.0–26.9)^*^
Systolic BP, mmHg	117.1 ± 17.4	125.4 ± 15.1^*^	119.1 ± 18.6	130.8 ± 18.0^*^
Diastolic BP, mmHg	71.8 ± 11.2	79.8 ± 9.2^*^	72.5 ± 11.0	79.4 ± 10.8^*^
Total cholesterol, mg/dl	213.9 ± 56.2	202.7 ± 57.4	230.1 ± 57.7	220.1 ± 55.4
LDL cholesterol, mg/dl	129.4 ± 44.4	115.8 ± 47.6^*^	148.3 ± 43.7	131.1 ± 46.7^*^
HDL cholesterol, mg/dl	48.7 ± 12.7	36.6 ± 10.2^*^	55.7 ± 10.6	45.0 ± 13.0^*^
Triglycerides, mg/dl	145 (106–233)	274 (201–387)^*^	113 (92–146)	198 (152–322)^*^
eGFR, ml/min/1.73m^2^	97.2 ± 16.8	96.0 ± 15.7	92.6 ± 14.7	90.4 ± 18.0
Uric acid, mg/dl	4.8 ± 1.3	5.3 ± 1.6^*^	4.3 ± 1.1	5.3 ± 1.8^*^
AST, U/L	28 (21–40)	28 (23–50)	21 (19–26)	24 (20–34)^*^
ALT, U/L	27 (18–51)	37 (23–63)^*^	20 (16–30)	27 (20–43)^*^
GGT, U/L	45 (29–107)	56 (33–98)	28 (23–45)	47 (28–84)^*^
Alkaline phosphatase, U/L	86 (65–105)	83 (68–102)	91 (71–106)	81 (63–103)
Glucose, mg/dl	90 (83–96)	109 (93–135)^*^	90 (83–95)	104 (92–128)^*^
HbA1c, mg/dl	5.4 ± 1.3	6.1 ± 1.6^*^	5.3 ± 0.8	5.8 ± 1.0^*^
HOMA-IR, mg/dl	1.5 (1.1–2.5)	3.0 (1.4–5.0)	1.6 (1.0–2.4)	3.0 (1.5–5.1)^*^

*Patients were classified as having central obesity if waist circumference was ≥80 cm in women, or ≥94 cm in men. MH, metabolically health (absence of metabolic syndrome); MUH, metabolically unhealthy (presence of metabolic syndrome); ART, antiretroviral therapy; PI, protease inhibitors; NNRTI, non-nucleoside reverse transcriptase inhibitors; NRTI, nucleoside reverse transcriptase inhibitors; BP, blood pressure; LDL, low density lipoproteins; HDL, high density lipoproteins; eGFR, estimated glomerular filtration rate; AST, aspartate transaminase; ALT, alanine transaminase; GGT, gamma-glutamyl transferase; HOMA-IR, homeostatic model assessment of insulin resistance*.

* *p < 0.05 vs. MH*.

### Association of anthropometric parameters with cardiometabolic comorbidities

The unadjusted and adjusted associations of anthropometric parameters with cardiometabolic comorbidities are presented in Table 5.

**Table 5:** Association of anthropometric parameters with cardiometabolic comorbidities.

	**Unadjusted association**	**Model 1**	**Model 2**	**Model 3**
**DIABETES MELLITUS**				
**BMI categories**				
Normal weight	(Reference)	(Reference)	(Reference)
Overweight	1.24 (0.80–1.92)	1.09 (0.69–1.72)	1.63 (0.96–2.76)
Obesity	1.56 (0.95–2.56)	1.63 (0.96–2.76)	1.57 (0.92–2.70)
**Waist circumference**				
Women < 80 cm/Men < 94 cm	(Reference)	(Reference)	(Reference)	(Reference)
Women ≥80 cm/Men ≥94 cm	1.39 (0.93–2.08)	**1.60 (1.03–2.50)**	1.57 (1.00–2.48)	1.30 (0.73–2.31)
**Waist-to-hip ratio**				
Women < 0.85/Men < 0.90	(Reference)	(Reference)	(Reference)	(Reference)
Women ≥0.85/Men ≥0.90	**3.43 (1.33–8.82)**	2.44 (0.92–6.48)	**2.79 (1.03–7.55)**	2.20 (0.79–6.08)
**Waist-to-height ratio**				
< 0.50	(Reference)	(Reference)	(Reference)	(Reference)
≥0.50	1.74 (0.99–3.06)	1.44 (0.80–2.61)	1.39 (0.76–2.52)	1.04 (0.52–2.07)
**HYPERTENSION**				
**BMI categories**				
Normal weight	(Reference)	(Reference)	(Reference)
Overweight	**2.25 (1.51–3.33)**	**1.97 (1.31–2.97)**	**2.03 (1.3–3.07)**
Obesity	**1.80 (1.14–2.85)**	**1.73 (1.07–2.80)**	**1.69 (1.03–2.75)**
**Waist circumference**				
Women < 80 cm/Men < 94 cm	(Reference)	(Reference)	(Reference)	(Reference)
Women ≥ 80 cm/Men ≥94 cm	**1.81 (1.26–2.58)**	**1.82 (1.21–2.74)**	**1.84 (1.22–2.79)**	1.41 (0.84–2.37)
**Waist-to-hip ratio**				
Women < 0.85/Men < 0.90	(Reference)	(Reference)	(Reference)	(Reference)
Women ≥0.85/Men ≥ 0.90	**1.94 (1.10–3.41)**	1.42 (0.78–2.56)	1.49 (0.82–2.72)	1.16 (0.62–2.18)
**Waist-to-height ratio**				
< 0.50	(Reference)	(Reference)	(Reference)	(Reference)
≥0.50	**2.62 (1.68–4.10)**	**2.10 (1.31–3.36)**	**2.13 (1.33–3.40)**	1.66 (0.96–2.87)
**DYSLIPIDEMIA**				
**BMI categories**				
Normal weight	(Reference)	(Reference)	(Reference)
Overweight	1.10 (0.45–2.71)	0.92 (0.37–2.30)	0.92 (0.36–2.30)
Obesity	1.12 (0.38–3.30)	1.02 (0.34–3.07)	0.99 (0.33–2.98)
**Waist circumference**				
Women < 80 cm/Men < 94 cm	(Reference)	(Reference)	(Reference)	(Reference)
Women ≥ 80 cm/Men ≥ 94 cm	1.24 (0.55–2.78)	1.14 (0.46–2.81)	1.14 (0.46–2.81)	1.05 (0.33–3.32)
**Waist-to-hip ratio**				
Women < 0.85/Men < 0.90	(Reference)	(Reference)	(Reference)	(Reference)
Women ≥ 0.85/Men ≥ 0.90	1.10 (0.24–4.99)	0.55 (0.12–2.64)	0.53 (0.11–2.53)	0.39 (0.07–2.06)
**Waist-to-height ratio**				
< 0.50	(Reference)	(Reference)	(Reference)	(Reference)
≥0.50	1.49 (0.58–3.82)	1.15 (0.43–3.08)	1.13 (0.42–3.02)	1.04 (0.33–3.32)
**METABOLIC SYNDROME**				
**BMI categories**	
Normal weight	(Reference)	(Reference)	(Reference)
Overweight	**3.69 (2.48–5.48)**	**3.43 (2.29–5.14)**	**3.74 (2.48–5.64)**
Obesity	**4.58 (2.80–7.51)**	**4.51 (2.72–7.48)**	**4.7 (2.82–7.93)**
**Waist circumference**				
Women < 80 cm/Men < 94 cm	(Reference)	(Reference)	(Reference)	(Reference)
Women ≥ 80 cm/Men ≥ 94 cm	**7.33 (4.97–10.80)**	**11.11 (6.91–17.87)**	**12.10 (7.45–19.66)**	**11.93 (6.63–21.4)**
**METABOLIC SYNDROME**				
**Waist-to-hip ratio**				
Women < 0.85/Men < 0.90	(Reference)	(Reference)	(Reference)	(Reference)
Women ≥ 0.85/Men ≥ 0.90	**6.65 (3.40–13.01)**	**5.78 (2.92–11.44)**	**5.50 (2.76–10.94)**	**3.63 (1.78–7.41)**
**Waist-to-height ratio**				
< 0.50	(Reference)	(Reference)	(Reference)	(Reference)
≥0.50	**8.10 (4.77–13.78)**	**7.47 (4.33–12.89)**	**7.44 (4.31–12.86)**	**4.62 (2.50–8.53)**
**FRAMINGHAM 10-y RISK** ≥**10%**				
**BMI categories**				
Normal weight	(Reference)	(Reference)	(Reference)
Overweight	**1.66 (1.13–2.44)**	0.85 (0.48–1.50)	0.85 (0.48–1.51)
Obesity	1.35 (0.86–2.13)	1.17 (0.60–2.30)	1.20 (0.61–2.37)
**Waist circumference**				
Women < 80 cm/Men < 94 cm	(Reference)	(Reference)	(Reference)	(Reference)
Women ≥ 80 cm/Men ≥ 94 cm	**1.45 (1.01–2.06)**	1.46 (0.84–2.53)	1.47 (0.84–2.56)	1.10 (0.54–2.25)
**Waist-to-hip ratio**				
Women < 0.85/Men < 0.90	(Reference)	(Reference)	(Reference)	(Reference)
Women ≥ 0.85/Men ≥ 0.90	**9.01 (3.77–21.6)**	**8.16 (2.52–26.38)**	**8.11 (2.50–26.32)**	**7.45 (2.26–24.55)**
**Waist-to-height ratio**				
< 0.50	(Reference)	(Reference)	(Reference)	(Reference)
≥0.50	**2.50 (1.54–4.08)**	1.34 (0.67–2.65)	1.35 (0.68–2.66)	0.99 (0.45–2.16)

Model 1, gender and age; Model 2, gender, age, and ART duration; Model 3, gender, age, ART duration, and BMI.

*The values highlighted in bold are statistically significant (P < 0.05)*.

In the unadjusted analysis, a high waist-to-hip ratio was significantly associated with the presence of diabetes. After adjustment for gender and age (Model 1), only increased waist circumference was associated with diabetes, while after simultaneous adjustment for gender, age and ART duration (Model 2), only a high waist-to-hip ratio was associated with the presence of diabetes. No anthropometric parameter was associated with diabetes in Model 3, after adjusting for gender, age, ART duration, and BMI.

With regards to the association with hypertension, BMI categories, both a high waist-to-hip ratio and a high waist-to-height ratio were associated with a higher prevalence of hypertension, even after adjustment for gender, age, and ART duration. The anthropometric parameter associated with higher odds for hypertension, after adjustment for gender, age, and ART duration, was the waist-to-height ratio (odds ratio [OR]: 2.13 [1.33–3.40] in model 2). After adjustment for BMI (Model 3), the waist circumference, waist-to-hip ratio and waist-to-height ratio were all no longer associated with hypertension.

No anthropometric parameter was associated with dyslipidemia in the unadjusted or adjusted models.

Metabolic syndrome was significantly more frequent with being overweight or obesity, when compared with normal weight, and also with a high waist-circumference, a high waist-to-hip ratio, and a waist-to-height ratio, both in the unadjusted and adjusted models. Waist circumference was the anthropometric parameter that was associated with the highest odds of metabolic syndrome (OR 12.10 [7.45–19.66] in Model 2; OR 11.93 [6.63–21.4] in Model 3).

There was a significant association between a Framingham 10 years risk score ≥10% and high waist circumference, high waist-to-hip ratio, high waist-to-height ratio, or being overweight in the unadjusted model. In the adjusted models, only a high waist-to-hip ratio was significantly associated with a Framingham 10 years risk score ≥10% (OR in the model 3: 7.45 [2.26–24.55]).

## Discussion

Our study highlights a high prevalence of overweight, obesity, and related comorbidities in patients infected with HIV. The prevalence of excessive weight and metabolic comorbidities progressively increased with age. Patients with obesity, as defined by BMI or abdominal circumference, showed a higher prevalence of cardiometabolic disturbances. Furthermore, when patients were divided according to metabolic health status, several clinical differences were present and a clear distinct cardiometabolic profile was observed among metabolically unhealthy patients, when compared with metabolically healthy patients, even when patients were divided according to BMI category or central obesity. Finally, our results showed that anthropometric parameters are significantly associated with cardiometabolic comorbidities and the Framingham 10 years risk score in patients with HIV.

The higher prevalence of obesity and cardiometabolic comorbidities with aging has been well characterized in the general population (50–52). However, the effect of HIV infection on such a relationship remains not so clear. Our results show that in patients with HIV, aging is also an important contributor for cardiometabolic dysfunction. It is important to highlight that even in younger patients (< 40 years), there was already a high incidence of cardiometabolic comorbidities. In our study, 10% of the patients in this age group had diabetes, 53.7% had hypertension, and 47.6% had metabolic syndrome. This prevalence is significantly higher than what is known for the Portuguese population (53). In our study, there was a significant increase in the prevalence of diabetes, hypertension, dyslipidemia, and metabolic syndrome with aging. Our results are in accordance with previous reports from HIV patients. Jericó et al. (54) describe a prevalence of 5.1% of metabolic syndrome among HIV-infected patients under 30 years old, and a prevalence of 27.0% in those aged 50–59 years old. In a meta-analysis of global HIV-infected population, patients with an age above the median (>41 years) compared with younger participants (≤ 41 years) had a higher prevalence of metabolic syndrome (19.7 vs. 13.2%, according to ATPIII-2001 criteria, and 22.3 vs. 16.4% according to IDF-2005 criteria) (55). In the general population, aging has been referred to as the most important determinant of cardiovascular health (56). Ageing promotes a progressive decline in numerous physiological processes, with relevant changes in cardiovascular tissues. Ageing is also associated with increased arterial stiffness, impaired endothelial function, and altered left ventricular diastolic and systolic function (56). Furthermore, aging potentiates mechanisms of lipid and glucose metabolism dysfunction (57). In patients with HIV, an elevated immune activation and the presence of persistent low-grade inflammation may also play an important role in further increasing the risk in this population (58).

The complex interaction between the immune system and HIV infection leads to increased cytokine expression and vascular damage (59). For example, Boccara et al. found that in naïve HIV-infected patients, increased plasma markers of endothelial dysfunction (ICAM1, ICAM2, ICAM3, and VCAM1) was already prevalent at diagnosis, without significant changes during follow-up (60). Moreover, endothelial function is also strictly connected with the development of early atherosclerosis (61). Even in patients who were not receiving antiretroviral therapy, manifest immunodeficiency or detectable viraemia, HIV infection has been associated with accelerated atherosclerosis (62). Hsue et al. also found that HIV-infected patients have a higher prevalence of atherosclerotic disease and vascular dysfunction when compared with age-matched uninfected patients (63). HIV infection has been associated with higher CRP levels when compared with healthy controls. Increased CRP levels are also associated with increased cardiovascular risk (62), which suggests that persistent inflammation contributes to vascular dysfunction and increased cardiovascular risk in HIV patients.

In our study, the prevalence of overweight and obesity was similar, regardless of the duration of infection. Patients in the normal weight group had a longer median period of ART than patients with overweight or obesity. Previously, a cross-sectional study with 862 patients with 21.5 years of HIV infection follow-up also concluded that the prevalence of overweight and obesity was not influenced by the duration of infection (64). A meta-analysis on the association of ART with overweight/obesity also did not find a significant association (64). In contrast, other studies showed a higher BMI in patients who received ART (65), with significant weight gain after starting ART (3, 66). Other risk factors for excess weight gain apart from ART are likely to be important in HIV patients, and these should also be considered, in order to avoid the development of comorbidities in this population.

The type of antiretroviral therapy being administered may have a central role in the development of metabolic dysfunction in patients with HIV (67). The newer antiretroviral drugs present a safer metabolic profile. Whereas protease inhibitors have been associated with a relevant risk of the development of dyslipidemia and lipodystrophy (68), integrase inhibitors have a minimal impact on lipid profile and do not appear to be associated with lipodystrophy (69). Furthermore, the use of antiretroviral drugs with different mechanisms of action may exert synergic anti-adipogenic effects (70). In a study on human subcutaneous adipocytes, the use of several different combination antiretroviral therapy was associated with a greater inhibitory effect on preadipocyte proliferation and differentiation than each drug individually (71).

As in the general population, overweight and obesity are associated with a large number of comorbidities in HIV-infected patients, including diabetes, hypertension, and other cardiovascular diseases (64). Our study confirms that the prevalence of metabolic syndrome and hypertension is high in HIV-infected patients, and that being overweight and obesity further increase this risk. Patients with central obesity also had a significantly higher prevalence of prediabetes and presented higher blood pressure, total cholesterol, and LDL cholesterol than those with normal waist circumference. Although, in our study, patients with central obesity had higher levels of HDL compared to patients with normal waist circumference, this was probably related to the higher proportion of women among patients with central obesity.

Gender may also influence BMI among patients living with HIV/AIDS. In our study, there was a higher proportion of women with BMI over 30 kg/m^2^ and in the group with central obesity. There are conflicting reports in the literature regarding the relationship between gender and changes in BMI following the initiation of ART (3, 72, 73). In some studies, gender was not significantly associated with being overweight and obesity in HIV patients, while others have shown a higher risk of obesity among women in HIV patients (74–76). Sex differences in the regulation and action of leptin (77), different states of immune activation (78), and the effects of sex steroids may explain the differences in BMI and obesity between genders in patients with HIV (78).

It was previously reported that obesity favors the recovery of CD4+ T-cell counts (79, 80, 64). Amorosa et al. reported that CD4 count >200 cells/mm3 was associated with obesity and being overweight (81). Although in our study no association between obesity and immune cell counts was found, its cross-sectional design does not allow one to evaluate the impact of excessive weight on immune reconstitution over time (64). The proportion of patients with HIV-1 RNA loads < 50 copies/mL was also similar between groups. Previous studies have suggested that, when compared with normal and overweight patients, those with obesity may have lower levels of plasma HIV RNA (54). Latently HIV-infected and adipose-resident CD4+ T cells within adipose tissue may lead to impaired lipid metabolism and storage (82). Furthermore, it has been shown that HIV-1 RNA is positively correlated with resting energy expenditure (83). However, in agreement with our results, it has been suggested that in developed countries, nowadays the energy expenditure changes associated with HIV are unlikely to contribute to significant weight loss (84). There was a lower proportion of patients with central obesity treated with NRTI in comparison with patients without central obesity. This was consistent with a study of 1,177 women undergoing ART with 10,754 person-years of follow-up, which found that NRTI exposure was associated with reduced odds of having greater BMI, when compared with both PI use and NNRTI use (85).

There is a growing concern that HIV contributes to the prevalence of renal insufficiency worldwide (86). In our study, we found that eGFR was significantly lower in the central obesity group. It is well known that obesity is a major risk factor for comorbid conditions such as diabetes and hypertension, which contribute to the development of chronic kidney disease, which could explain this association (87).

It has been postulated that HIV infection can induce hepatic steatosis, either through the effects of immune activation on insulin tolerance and lipogenesis, or indirectly—through metabolic abnormalities and toxicity of antiretroviral therapy. However, a report from the Multicenter AIDS Cohort Study found that HIV-infected men had a lower risk of hepatic steatosis when compared to men without HIV infection (88). Central obesity is associated with increased risk of hepatic steatosis and higher levels of serum aminotransferases in the general population (89). The same association has been observed among patients with HIV (90). In our study, patients with normal waist circumference had higher levels of AST and ALT compared to patients with central obesity, although the difference was small. This unexpected result may be explained by the higher proportion of patients in the normal waist circumference group treated with NRTI, which is associated with hepatic toxicity (91).

A state of metabolically healthy obesity (MHO) has been described in the general population (92, 93). MHO is obesity without overtly-associated cardiometabolic disease, and has been variably defined to include people with BMI ≥25 or ≥30 kg/m^2^, and most commonly, ≤ 2 metabolic syndrome criteria (45, 94, 95). The prevalence of MHO varies according to the population and the definition used. There is currently no consensus criterion for the definition of MHO, and there is also a controversial debate as to whether MHO exists at all (19, 96). However, some studies have demonstrated increased mortality rates, increased cardiovascular disease risk, and differences in body composition and fat distribution among people diagnosed as having metabolically unhealthy obesity (MUHO) criteria (15, 97, 98). Numerous possible mechanisms underlying MUHO have been suggested, including adipose tissue distribution and inflammation. People with MHO may have less visceral fat and systemic inflammation and more favorable immune profiles than the metabolically unhealthy obese (99–102), suggesting that a spectrum of metabolic health can co-exist with obesity.

Another discussion is as to whether MHO exists among HIV-infected people. Given that HIV infection and ART are associated with a variety of metabolic changes, and the fact that the prevalence of the metabolic syndrome in HIV-infected people is high (24) and also that immunologic dysregulation and enhanced inflammation may exist independent of obesity in treated and untreated HIV-infected people, it is possible that HIV infection and obesity have a synergistic and detrimental effect on metabolic health, which could prevent the existence of MHO in these HIV-infected patients. In our study, most patients who were overweight and with obesity were classified as being metabolically unhealthy (71.0 and 75.2% respectively), whereas most patients in the normal BMI group were metabolically healthy (39.8% metabolically unhealthy participants in the normal BMI group), showing the impact of obesity *per se* in metabolic derangements. Furthermore, among patients with normal BMI, metabolically unhealthy patients were older and had a longer duration of HIV infection. Our results show that BMI has a great impact on metabolic health status. On the other hand, an older age and duration of HIV are also associated with the reduced likelihood of metabolic health among HIV-infected patients.

Patients with HIV present not only a high prevalence of obesity and increased waist circumference, but also significant changes of body composition and adipose tissue redistribution (103, 104). We have previously described that patients with HIV and lipodystrophy present an increase in total fat mass and upper-limbs fat mass, with a decrease in total, trunk, and lower-limbs fat-free mass over a follow-up period of 2 years (105). Those patients predominantly presented a central fat mass gain, with no changes in lower limbs fat mass, suggesting that peripheral adipocytes lose their regenerative capacity. We also described that HIV patients with lipodystrophy defined by fat mass ratio present a high prevalence of glucose disturbances and insulin resistance (106) and increased carotid intima-media thickness (107), which suggests that adipose tissue redistribution is also a central factor for the prevalence of cardiovascular risk among HIV-infected patients. The volume of epicardial adipose tissue may also contribute to cardiovascular risk in patients with HIV. Guaraldi et al. described an association of epicardial adipose tissue with traditional risk factors for atherosclerosis and coronary artery calcium (108). Furthermore, we have shown that fat redistribution is associated with adipokines profile. Fat reduction in HIV lipoatrophy is associated with low leptin levels, while visceral fat accumulation is mainly associated with decreased plasma adiponectin levels (109). Gast et al. also described that visceral adipose tissue contributes beyond overall adiposity to subclinical atherosclerosis, particularly among women (110).

In our results, both in the normal weight and overweight groups, metabolically unhealthy patients presented higher waist circumference, waist-to-hip ratio, and waist-to-height ratio. In the obesity group, metabolically unhealthy participants had lower hip circumference and higher waist-to-hip ratio. The proportion of MUHO patients was significantly higher in the group with central obesity, when compared with participants with normal waist circumference (77.2 vs. 31.6, *p* < 0.001). These results highlight that this specific fat redistribution with high visceral fat accumulation, when compared with subcutaneous fat depots, may play a major role in the pathogenesis of MUHO and cardiometabolic risk. Inflammation in adipose tissue has been proposed as being another key factor that explains the metabolic alterations associated with obesity (111).

Unquestionably, obesity is associated with inflammation and is characterized by a low-level chronic inflammatory state (112). However, there is controversy regarding the presence of an inflammatory status in patients with MHO, although most of the studies were carried out in the non-HIV-infected population. Phillips and Perry (18) demonstrated that individuals with MHO presented a more favorable inflammatory status than their metabolically unhealthy peers, who presented a reduced white blood cell count, higher adiponectin levels, and lower concentrations of: complement component-3; C-reactive protein; tumor necrosis factor α; interleukin 6, and plasminogen activator inhibitor 1. On the contrary, other studies have shown that circulating concentrations of proinflammatory factors may be increased in both the MHO and MUHO groups (113, 114).

Our results show that waist circumference, waist-to-hip ratio, waist-to-height ratio, and BMI categories are all associated with cardiometabolic comorbidities, although the type of cardiometabolic comorbidity and the strength of the association vary with the anthropometric parameter. In our study, a high waist circumference was the parameter most associated with metabolic syndrome, whereas a high waist-to-height ratio was the anthropometric parameter most associated with the prevalence hypertension, and a high waist-to-hip ratio was the best anthropometric predictor of diabetes mellitus and a Framingham 10 years risk score ≥10%. In our study, no anthropometric parameter was associated with dyslipidemia. This lack of association is probably related to the strict definition for dyslipidemia in HIV-patients that we used, leading to a high prevalence of dyslipidemia across the patients under study, and a reduction in the ability to detect differences according to anthropometric parameters.

Furthermore, in our study, all anthropometric parameters were significantly associated with metabolic syndrome, highlighting the importance of obesity in the development of metabolic syndrome. The finding that waist circumference is the parameter most associated with the metabolic syndrome is probably related to the fact that waist circumference is also one of the parameters used to define the metabolic syndrome. The strong association of hypertension with waist-to-height ratio is consistent with previous studies of the general population of different ethnicities (115–117). Choi JR et al. reported that waist-to-height ratio was a better predictor of incident hypertension than waist-to-hip ratio, or BMI (117). In a population-based study of Brazilian women (116), waist-to-height ratio was also the best anthropometric predictor of prevalent hypertension. On the other hand, in our study, a high waist-to-hip ratio was the best predictor of prevalent diabetes mellitus and of a Framingham 10 years risk score above 10%. A recent mendelian randomisation study of the general population (118) showed that a polygenic risk score for increased waist-to-hip ratio adjusted for body mass index was associated with adverse cardiometabolic traits and higher risks for both type 2 diabetes and coronary heart disease, which supports a causal association between high waist-to-hip ratio and type 2 diabetes and cardiovascular risk. In agreement with previous studies of HIV patients (119–121), our results also highlight that in this population, waist-hip ratio is a better marker of cardiometabolic comorbidities and risk of cardiovascular events than the measurement of BMI.

We have to acknowledge some limitations of our study. Due to the cross-sectional design of our study, we cannot draw conclusion regarding causality of the observed associations. Furthermore, as we did not work with an HIV-uninfected control group, we cannot make conclusions about the relative importance of HIV infection or antiretroviral drugs on metabolic comorbidities, body composition, and cardiovascular risk. Finally, we do not have detailed information regarding previous therapeutic regimens, and over the years, patients may have been subject to different therapeutic regimens, which may also have contributed to their metabolic and anthropometric profile.

Our study has several strong points. We evaluated the effects of obesity and body composition on metabolic comorbidities and cardiovascular risk of patients with HIV, taking into account the effects of aging, the characterization of patients as metabolically healthy, or metabolically unhealthy, and also the different anthropometric parameters. Although the association of body composition and metabolic status with metabolic comorbidities and cardiovascular risk is well described for the general population, these associations were not so well characterized for HIV patients. Our study significantly contributed to further understanding the role of obesity, metabolic health status, and different anthropometric parameters for the metabolic comorbidities and cardiovascular risk of patients with HIV. All patients were evaluated according to a strict protocol which included detailed clinical, anthropometric, and metabolic parameters. Furthermore, this study was performed in a hospital unit that is highly experienced in the assessment of metabolic and body fat abnormalities in HIV-infected patients.

Our findings can be summarized as follows: (1) being overweight, obesity and abdominal obesity increased significantly with age, as did associated metabolic comorbidities, such as diabetes, dyslipidemia, hypertension, and metabolic syndrome; (2) patients with obesity defined by BMI and by central obesity had more metabolic alterations (hypertension, hyperuricemia, fasting plasma glucose levels) and a greater level of metabolic syndrome and CV risk evaluated by the 10 year Framingham risk score, when compared with those with normal weight; (3) metabolically healthy individuals were present in the subpopulation of normal weight, although most patients were metabolically unhealthy in the overweight and obesity groups; (4) similarly, among patients without central obesity, there was a significant proportion of metabolically healthy participants, while among patients with central obesity, most patients were metabolically unhealthy; (5) after adjustment for gender and age, only increased waist circumference was associated with diabetes, while after simultaneous adjustment for gender, age, and ART duration, only high waist-to-hip ratio was associated with the presence of diabetes. The anthropometric parameter associated with the highest odds for hypertension was waist-to-height ratio, after adjustment for gender, age, and ART duration. Waist circumference was the anthropometric parameter that was most associated with the highest odds of metabolic syndrome. In the adjusted models, only high waist-to-hip ratio was significantly associated with a Framingham 10 years risk score ≥10%.

In conclusion, patients with HIV present a high prevalence of being overweight, obesity, and related comorbidities. Ageing is an important contributor for excessive weight and metabolic dysfunction in this population. The prevalence of metabolic comorbidities is significantly higher in patients with high BMI and with central obesity. Although some patients were classified as being metabolically healthy, both in the normal weight and overweight groups, there was a significantly higher proportion of metabolically unhealthy patients among groups with excessive weight and central obesity, and those classified as being metabolically unhealthy presented a significantly higher cardiovascular risk. From a clinical point of view, our results highlight the importance of systematically evaluating and addressing obesity, metabolic comorbidities, and cardiovascular risk in patients infected by HIV.

## Ethics statement

This study was carried out in accordance with the recommendations of institutional and national guidelines and regulations, with written informed consent from all subjects. All subjects gave written informed consent in accordance with the Declaration of Helsinki. The protocol was approved by the Ethics Committee of São João Hospital Center.

## Data availability statement

The datasets for this manuscript are not publicly available, as it was stated in the consent form signed by participants that all data are confidential and will only be made available to the researchers. Requests to access the datasets should be sent to paula_freitas@sapo.pt.

## Author contributions

JN, VG, and PF conceived the study and participated in its design. PF participated in the acquisition of data. JN performed the statistical analysis. All authors contributed to the interpretation of the data for the study, critically revised the work, and also approved the final version of the manuscript.

### Conflict of interest statement

The authors declare that the research was conducted in the absence of any commercial or financial relationships that could be construed as a potential conflict of interest.
